# Functional Crosstalk between CB and TRPV1 Receptors Protects Nigrostriatal Dopaminergic Neurons in the MPTP Model of Parkinson's Disease

**DOI:** 10.1155/2020/5093493

**Published:** 2020-09-28

**Authors:** Rayul Wi, Young Cheul Chung, Byung Kwan Jin

**Affiliations:** ^1^Department of Neuroscience, Graduate School, School of Medicine, Kyung Hee University, Seoul 02447, Republic of Korea; ^2^Department of Biochemistry & Molecular Biology, School of Medicine, Kyung Hee University, Seoul 02447, Republic of Korea

## Abstract

The present study examined whether crosstalk between cannabinoid (CB) and transient potential receptor vanilloid type 1 (TRPV1) could contribute to the survival of nigrostriatal dopamine neurons in the 1-methyl-4-phenyl-1,2,3,6-tetrahydropyridine (MPTP) mouse model of Parkinson's disease (PD). MPTP induced a significant loss of nigrostriatal dopamine neurons and glial activation in the substantia nigra (SN) and striatum (STR) as visualized by tyrosine hydroxylase (TH) or macrophage antigen complex-1 (MAC-1) or glial fibrillary acidic protein (GFAP) immunocytochemistry, respectively. RT-PCR analysis shows the upregulation of inducible nitric oxide synthase, interleukin-1*β*, and tumor necrosis factor-*α* in microglia in the SN in vivo, indicating the activation of the inflammatory system. By contrast, treatment with capsaicin (a specific TRPV1 agonist) increased the survival of dopamine neurons in the SN and their fibers and dopamine levels in the STR in MPTP mice. Capsaicin neuroprotection is accompanied by inhibiting MPTP-induced glial activation and production of inflammatory cytokines. Treatment with AM251 and AM630 (CB1/2 antagonists) abolished capsaicin-induced beneficial effects, indicating the existence of a functional crosstalk between CB and TRPV1. Moreover, treatment with anandamide (an endogenous agonist for both CB and TRVP1) rescued nigrostriatal dopamine neurons and reduced gliosis-derived neuroinflammatory responses in MPTP mice. These results suggest that the cannabinoid and vanilloid system may be beneficial for the treatment of neurodegenerative diseases, such as PD, that are associated with neuroinflammation.

## 1. Introduction

Parkinson's disease (PD) is a well-known neurodegenerative disorder that is characterized by the degeneration of dopamine neurons in the substantia nigra pars compacta (SNpc) and dopamine deficiency in the striatum (STR), consequently resulting in motor dysfunction [1, 2]. Several lines of clinical and experimental evidence suggest that proteinopathy related to *α*-synuclein, environmental toxins, mitochondrial dysfunction, and oxidative stress is associated with the molecular mechanisms of PD etiology [3–5]. Among them, gliosis-derived neuroinflammation is a considerable part of PD development [3, 5]. Recent evidence has shown that reactive microglia and astrocytes are known to play a crucial role in the production of proinflammatory mediators such as nitric oxide (NO), inducible nitric oxide synthase (iNOS), myeloperoxidase, and proinflammatory cytokines [5–7]. These inflammatory mediators are attributable to the degeneration of nigrostriatal dopamine neurons in animal models of PD and PD patients [3, 5, 7]. Current experimental studies, such as the development of neuroprotective agents on dopamine neurons through regulating glial activation and preventing production of neurotoxic inflammatory molecules, have provided opportunities to develop innovative strategies for PD therapy [5].

Transient receptor potential vanilloid subtype 1 (TRPV1), a polymodal and nonselective cation channel, is activated by a number of endogenous and exogenous stimuli, including natural vanilloids (capsaicin and resiniferatoxin), heat, acids, and endocannabinoids such as anandamide (AEA) [8, 9]. TRPV1 is widely present in various neurons and glial cells (microglia and astrocytes) in the nigrostriatal pathway *in vivo* [10–12]. Many experimental studies demonstrated that TRPV1 activation by capsaicin (CAP) prevents the degeneration of nigrostriatal dopamine neurons in the 1-methyl-4-phenylpyridinium- (MPP^+^-) or 1-methyl-4-phenyl-1,2,3,6-tetrahydropyridine- (MPTP-) or 6-hydroxy dopamine (6-OHDA-) lesioned rodent model of PD via inhibiting glial-derived inflammatory responses and producing ciliary neurotrophic factor (CNTF) [11–13]. We recently demonstrated that TRPV1 activation by CAP increased the survival of nigral dopamine neurons by modulating the M1/M2 microglia/macrophage phenotype in lipopolysaccharide- (LPS-) injected SN [10], indicating that TRPV1 is a possible therapeutic target to treat PD.

Due to the presence of an intracellular binding site for AEA [8] and colocalization of TRPV1 and CB receptors *in vivo* and *in vitro* [14, 15], TRPV1 is thought to be a possible ionotropic receptor counterpart for cannabinoid (CB) receptors. A pharmacological study has reported that both TRPV1 and CB1 are involved with AEA-mediated neuroprotection in the *in vivo* rat model of ouabain-induced excitotoxicity [16] and pentylenetetrazole-induced seizures [17]. However, crosstalk between TRPV1 and CB receptors that possibly influence the fate of dopamine neurons in PD was not determined yet. The present study investigates functional interactions between these two receptors, which might contribute to the survival of dopamine neurons by regulating gliosis-derived neuroinflammation in the MPTP-intoxicated PD mouse model *in vivo*.

## 2. Materials and Methods

### 2.1. Chemicals

The following chemicals were purchased from the following companies: AEA, AM251, and AM630 were purchased from Tocris Bioscience, Bristol, UK, and CAP and MPTP were purchased from Sigma-Aldrich, St. Louis, MO, USA. AEA, AM251, and AM630 were dissolved in 1% dimethyl sulfoxide and then diluted with sterile phosphate-buffered saline (PBS). CAP were dissolved in ethanol and Tween-80 and then diluted with phosphate-buffered saline (PBS; 1 : 1 : 8, ethanol : Tween-80 : PBS).

### 2.2. Animals and Drug Treatment

**Figure 1:**
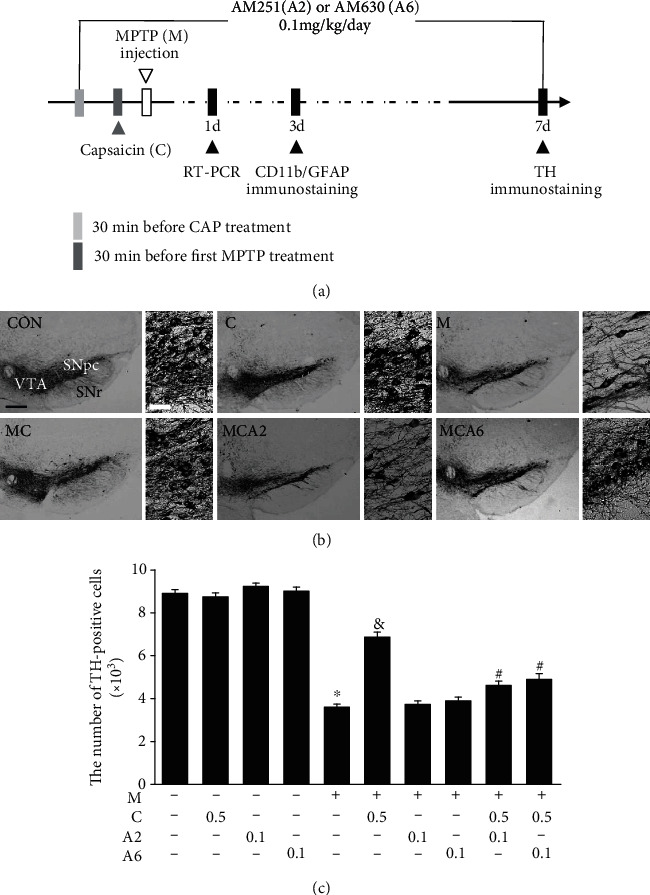
The CB receptor is associated with TRPV1-activated neuroprotection in the SN of MPTP-treated mice in vivo. (a) Diagram of the experimental design. Mice were intraperitoneally given an injection of PBS or MPTP. All mice intraperitoneally received PBS as a control or cannabinoid (CB) antagonist (AM251 (A2) or AM630 (A6); 0.1 mg/kg/day) for 7 days at 30 min before capsaicin (C) and 1 hour before MPTP and a single injection of capsaicin (0.5 mg/kg) at 30 min before MPTP. Mice that received PBS as a control (CON); capsaicin (C); MPTP (M); MPTP and capsaicin (MC); MPTP, capsaicin, and AM251 (MCA2); or MPTP, capsaicin, and AM630 (MCA6) were sacrificed at 7 days after the last MPTP injection. Brains were removed, and coronal sections (30 *μ*m) were cut using a sliding microtome. Every sixth serial section was selected and processed for TH immunohistochemical staining. (b) Photomicrographs of TH^+^ neurons in the SN. Higher magnification of each group for TH staining, respectively. These data are representative of five to six animals per group. (c) TH^+^ neurons were counted using a stereological technique in the SN. Bars represent the means ± SEM of five to six animals per group. ^∗^*P* < 0.001, significantly different from control. ^&^*P* < 0.001, significantly different from MPTP. ^#^*P* < 0.001, significantly different from MPTP and capsaicin (one-way ANOVA with the Neuman-Keuls post hoc test). SNpc: substantia nigra pars compacta; SNr: substantia nigra pars reticulata; VTA: ventral tegmental area. Scale bars: 250 *μ*m (left panel for (b)). Scale bars: 50 *μ*m (right panel for (b)).

**Figure 2:**
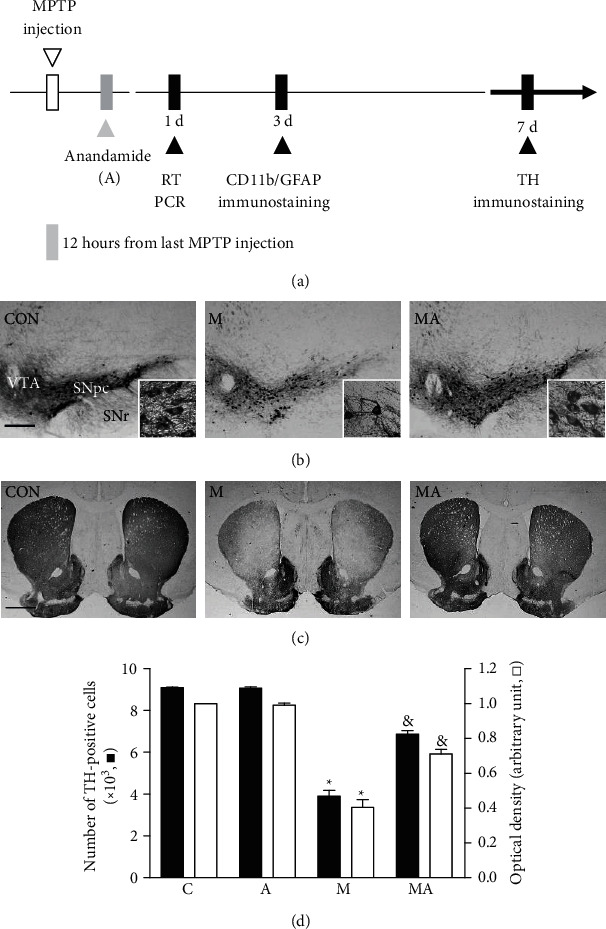
Anandamide prevents MPTP-induced degeneration of dopamine neurons in vivo. (a) Diagram of the experimental design. Mice were intraperitoneally given an injection of PBS as a control (CON) or MPTP only (M) or MPTP + anandamine (0.5 mg/kg) 12 hours after the last injection of MPTP (MA) and were sacrificed 7 days after the last MPTP injection. (b, c) Photomicrographs of TH^+^ neurons in the SN (b) and TH^+^ fibers in the striatum (c). Insets show higher magnifications of (b) and (c), respectively. (d) Number of TH^+^ neurons in the SN pars compacta (SNpc) and optical density of TH^+^ fibers in the striatum. Data are presented as means ± SEM of six animals per group. C: PBS-treated control; A: anandamide; M: MPTP; MA: MPTP and anandamide; SNpc: substantia nigra pars compacta; VTA: ventral tegmental area. ^∗^*P* < 0.001, significantly different from control. ^&^*P* < 0.001, significantly different from MPTP only (one-way ANOVA with the Neuman-Keuls post hoc test). Scale bars: (b) 300 *μ*m; (c) 500 *μ*m.

All experiments were conducted in accordance with the approved animal protocols and guidelines established by Kyung Hee University (KHUASP(SE)-10-030). As previously described [11, 18–21], C57BL/6 mice (eight-ten-week-old male mice) received four intraperitoneal injections of PBS or MPTP (20 mg/kg, free base; Sigma-Aldrich) dissolved in saline at 2-hour intervals. For the experimental group, mice received a single injection of CAP (0.5 mg/kg) into the peritoneum 30 min before the MPTP injections. To inhibit activation of CB receptors, animals received AM251 (CB1 receptor antagonist, 0.1 mg/kg) and AM630 (CB2 receptor antagonist, 0.1 mg/kg) into the peritoneum 30 min before treatment with CAP each day for 7 days (Figure 1(a)). For another experimental group, mice received a single injection of PBS as a control or AEA (0.5 mg/kg) after 12 hours from the last MPTP injection (Figure 2(a)).

### 2.3. Tissue Preparation and Immunohistochemistry

As previously described [11, 18, 19], animals were perfused with saline solution (0.9%) containing heparin (1,000 units/l) and fixed with 4% paraformaldehyde solution in 0.1 M phosphate-buffered solution. Brain tissues were cryoprotected, and then, 30 *μ*m sections were cut. Tissues were rinsed with PBS and then incubated with PBS containing 0.5% BSA and each of the following primary antibodies: rabbit anti-tyrosine hydroxylase (TH; 1 : 2000, Pel-Freez, Brown Deer, WI, USA) for dopamine neuron, rabbit anti-glial fibrillary acidic protein (GFAP; 1 : 5000, Neuromics, Edina, MN, USA) for astrocytes, and rat anti-CD11b (CD11b; 1 : 200, AbD Serotec, Oxford, UK) for microglia/macrophages. The following day, the brain sections were incubated with the appropriate secondary antibodies (biotinylated anti-rabbit or anti-rat antibody (1 : 400, KPL, Milford, MA, USA)) and developed with 3,3′-diaminobenzidine (DAB; Sigma-Aldrich) and 0.003% hydrogen peroxide in 0.1 M PBS.

### 2.4. Stereological Cell Counts

As described previously [11, 18, 19], midbrain sections were stained with TH and the total number of TH-positive neurons in the SNpc was counted in each animal group at 7-day postinjection (MPTP or saline) using the optical fractionator method performed on an Olympus Computer-Assisted Stereological Toolbox System version 2.1.4 (Olympus Danmark A/S, Ballerup, Denmark). To estimate the distribution of TH^+^ cells in the region of interest, the region of interest was defined and then, the area of interest was precisely marked at low magnification. The total number of neurons was estimated according to the optical fractionator equation. More than 300 points over all sections of each specimen were analyzed.

### 2.5. Densitometric Analysis

The measurement of the optical density (OD) of the TH^+^ fiber in the STR was performed with the Image-Pro Plus system (Version 4.0; Media Cybernetics Inc., Silver Spring, MD) on a computer attached to a light microscope (Zeiss Axioskop, Oberkochen, Germany), as previously described [11, 18, 19]. To control variations in background illumination, the average of background density readings from the corpus callosum was subtracted from that of the density of the STR for each section (an average of 6 coronal sections of STR in each group). The average of all sections in each animal group was calculated separately, and then, it was statistically processed.

### 2.6. Real-Time PCR

Animals in each experimental group were sacrificed at the indicated time point (Figures 1(a) and 2(a)), and the bilateral SN regions were immediately isolated. The SN tissues were processed for real-time PCR, as previously described [11, 18, 19]. Total RNA was prepared with RNAzol B (Tel-Test Inc., Friendwood, TX, USA), and reverse transcription was carried out with the SuperScript II Reverse Transcriptase (Life Technologies, Rockville, MD, USA) according to the manufacturer's instructions. The primer sequences used in this study were as follows: 5′-TGGACCTTCCAGGATGAGGACA-3′ (forward) and 5′-TTCATCTCGGAGCCTGTAGTG-3′ (reverse) for IL-1*β*; 5′-GCGACGTGGAACTGGCAGAAGAG-3′ (forward) and 5′-TGAGAGGGAGGCCATTTGGGAAC-3′ (reverse) for TNF-*α*; 5′-GAGACAGGGAAGTCTGAAGCAC-3′ (forward) and 5′-CCAGCAGTAGTTGCTCCTCTTC-3′ (reverse) for iNOS; and 5′-CATCACTGCCACCCAGAAGACTG-3′ (forward) and 5′-ATGCCAGTGAGCTTCCCGTTCAG-3′ (reverse) for GAPDH. Real-time PCRs were performed in a reaction volume of 20 ml, which included 1 ml of a reverse transcription product as a template, 10 ml of the SYBR Green PCR Master Mix (Applied Biosystems, Warrington, UK), and 20 pmol of each primer described above. The PCR amplifications were performed with 40 cycles of 95°C for 30 s and 60°C for 60 s using ABI 7500 (Applied Biosystems). Average threshold cycle (*C*_T_) values of IL-1*β* and TNF-*α* from triplicate PCR reactions were normalized from average *C*_T_ values of GAPDH.

### 2.7. Analysis for Microglia Morphology

To analyze the degree of microglial activation in MPTP-treated SN with the absence or presence of CAP and/or CB1/2 antagonists, images were obtained from the same area in each tissue sample using a light microscope (Zeiss Axioskop, Oberkochen, Germany) with 40x magnification (1024 × 1024 pixels). As previously described [22], all original images were collected, converted to 8-bit grayscale, filtered to further increase the contrast of images, and skeletonized using ImageJ. Then, the skeletonized images were selected, and the information for the branch length signal was analyzed using the plugin AnalyzeSkeleton (2D/3D).

### 2.8. Statistics

All values are expressed as the mean ± s.e.m.. Statistical significance (*P* < 0.05 for all analysis) was assessed by the one-way ANOVA Newman-Keuls analyses using the Instat 3.05 software package (GraphPad Software, San Diego, CA, USA).

## 3. Results

### 3.1. CB Receptor Contributes to TRPV1-Activated Neuroprotection in the Nigrostriatal Dopamine Pathway of MPTP-Treated Mice *In Vivo*

**Figure 3:**
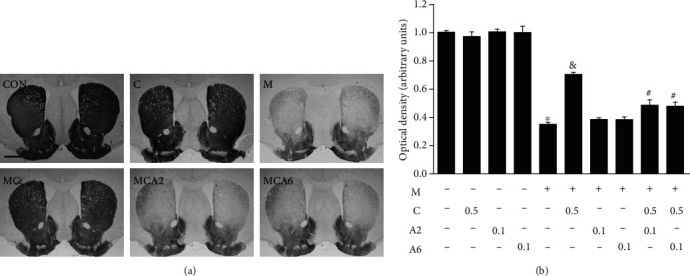
CB receptor is associated with TRPV1-activated neuroprotection in the striatum of MPTP-treated mice in vivo. The striatal tissues obtained from the same animals as those used in Figure 1 were immunostained with TH antibody for dopamine fibers. CON: PBS as control; C: capsaicin; M: MPTP; MC: MPTP and capsaicin; MCA2: MPTP, capsaicin, and AM251; MCA6: MPTP, capsaicin, and AM630. (a) Photomicrographs of TH^+^ fibers in the striatum. These data are representative of five to six animals per group. (b) The optical density of TH^+^ fibers in the striatum. Bars represent the means ± SEM of five to six animals per group. ^∗^*P* < 0.001, significantly different from control. ^&^*P* < 0.001, significantly different from MPTP. ^#^*P* < 0.001, significantly different from MPTP and CAP (one-way ANOVA with the Neuman-Keuls post hoc test). Scale bars: 500 *μ*m.

To examine the functional interactions between the CB receptor and TRPV1 in PD, we chose a mouse MPTP model of PD. Consistent with our previous reports [11, 18, 21], TH immunohistochemical analysis reveals the significant loss of TH^+^ cells in the SN (Figure 1(b)) and TH^+^ fibers in the STR (Figure 3(a)) in MPTP-treated mice compared to PBS-treated control mice (Figures 1(b) and 3(a)). The number of TH^+^ cells as assessed by stereology in the SN and the density of TH^+^ fibers in the STR are significantly reduced by 61% (Figure 1(c); *P* < 0.001) and 63% (Figure 3(b); *P* < 0.001), respectively, in MPTP-lesioned mice compared to PBS-treated control mice.

To determine the effects of TRPV1 activation on dopamine neurons, mice received intraperitoneally a single injection of the TRPV1 agonist capsaicin (CAP; 0.5 mg/kg) or vehicle at 30 min before MPTP treatment (Figure 1(a)). Similar to our previous reports [11, 23], pretreatment with CAP significantly increased the number of TH^+^ cells by 35% in the SN (Figure 1(c); *P* < 0.001) and increased the density of TH^+^ fibers by 40% in the striatum (Figure 3(b); *P* < 0.001) in MPTP-lesioned mice compared to MPTP-lesioned vehicle-treated mice. Pretreatment with CAP only as a control had no effects on TH^+^ cells (Figures 1(b) and 1(c)) and TH^+^ fibers (Figures 3(a) and 3(b)).

As a cannabinoid (CB) receptor can functionally interact with TRPV1 [15, 16], we wondered if the CB receptor could be associated with TRPV1-activated neuroprotection on nigrostriatal dopamine neurons *in vivo*. To test this, we inhibited the activation of the CB receptor using the CB1 antagonist AM251 and the CB2 receptor antagonist AM630. Mice intraperitoneally received the CB antagonists (AM251 or AM630; 0.1 mg/kg) or PBS each day for 7 days starting at 30 min before CAP treatment and 1 hour before MPTP treatment (Figure 1(a)). Pretreatment with AM251 and AM630 inhibited CAP neuroprotection against MPTP toxicity, resulting in a significant reduction of the number of TH^+^ cells in the SN (Figure 1(c); *P* < 0.001) and a reduction of the density of TH^+^ fibers in the striatum (Figure 3(b); *P* < 0.001) compared to the MPTP- and CAP-treated group. By contrast, pretreatment with CB antagonists did not prevent MPTP-induced degeneration of dopamine neurons in the absence of CAP treatment (Figures 1(c) and 3(b)). These results indicate that the CB receptor might regulate TRPV1-activated neuroprotection in MPTP-lesioned mice.

### 3.2. Interaction between CB Receptor and TRPV1 Regulates Proinflammatory Responses and Glial Activation *In Vivo*

**Figure 4:**
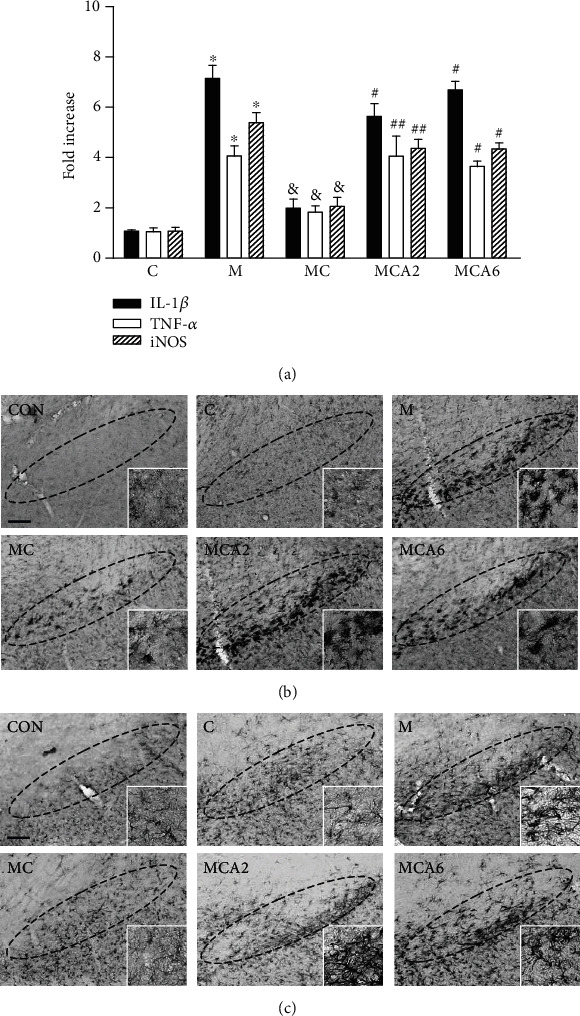
Crosstalk between CB and TRPV1 inhibits glial activation and expression of proinflammatory cytokines in the SN of MPTP-treated mice in vivo. Mice were intraperitoneally given an injection of PBS or MPTP. All mice intraperitoneally received vehicle as controls or cannabinoid (CB) antagonist (AM251 or AM630; 0.1 mg/kg/day) for 1 day or 3 days at 30 min before capsaicin (C) and 1 hour before MPTP and a single injection of capsaicin (0.5 mg/kg) at 30 min before MPTP. (a) Real-time PCR analysis showing mRNA expression of proinflammatory mediators (IL-1*β*, TNF-*α*, and iNOS) in the SN. Mice were sacrificed, and the total RNA was isolated from SN one day after the last injection of MPTP or vehicle in the absence or presence of CAP and CB1/2 antagonists (AM251 or AM630) (refer to Figure 1(a)). Bars represent the means ± SEM of four samples. C: control; M: MPTP; MC: MPTP and capsaicin; MCA2: MPTP, capsaicin, and AM251; MCA6: MPTP, capsaicin, and AM630. ^∗^*P* < 0.01, significantly different from control. ^&^*P* < 0.01: significantly different from MPTP. ^#^*P* < 0.01 and ^##^*P* < 0.05, significantly different from MPTP and capsaicin (one-way ANOVA with the Neuman-Keuls post hoc test). (b, c) Photomicrographs of CD11b^+^ microglia and GFAP^+^ astrocytes in the SN of MPTP-treated mice in vivo. Mice that received PBS as a control (CON); capsaicin (C); MPTP (M); MPTP and capsaicin (MC); MPTP, capsaicin, and AM251 (MCA2); or MPTP, capsaicin, and AM630 (MCA6) were sacrificed 3 days after the last MPTP injection (refer to Figure 1(a)). Brains were removed and coronal sections (30 *μ*m) were cut using a sliding microtome. Every sixth serial section was selected and immunostained with CD11b antibody for microglia (b) or GFAP antibody for astrocytes (c). Insets show higher magnifications of (b) and (c), respectively. These data are representative of five to six animals per group. Dotted lines indicate SNpc. Scale bars: (a) 300-500 *μ*m; (b) 250-420 *μ*m.

As TRPV1 activation by CAP prevents the expression of proinflammatory molecules such as interleukin-1*β* (IL-1*β*), tumor necrosis factor-*α* (TNF-*α*), and iNOS, which can impose neurotoxicity on dopamine neurons in MPTP-lesioned mice [10, 11], we wondered if its anti-inflammatory effect could be associated with the CB1 and CB2 receptors in the MPTP model. Consistent with our previous work [11], analysis by real-time PCR showed that CAP efficiently decreased MPTP-induced proinflammatory responses in the SN (Figure 4(a)). These anti-inflammatory effects of CAP were significantly reversed by the treatment of AM251 and AM630, indicating interactions between TRPV1 and the CB1/2 receptors.

As the activation of glial cells such as microglia and astrocytes is involved in neurodegeneration in MPTP mice [3, 11, 18, 21], we next investigated whether interactions between the two receptors, TRPV1 and the CB receptor, could regulate glial activation in the SN *in vivo*. To test this, we performed immunostaining with the CD11b antibody for microglia and the GFAP antibody for astrocytes. In the MPTP-treated group, numerous CD11b^+^-activated microglia (Figure 4(b)) and GFAP^+^-reactive astrocytes (Figure 4(c)) were observed in the SN *in vivo* compared to PBS control (Figures 4(b) and 4(c)). In line with our recent reports [11, 18], TRPV1 activation by CAP suppressed microglial activation (Figure 4(b)) and astroglial activation (Figure 4(c)). These inhibitory effects of CAP on glial activation was reversed by treatment with AM251 and AM630 *in vivo* (Figures 4(b) and 4(c)). CB1 and CB2 antagonists alone had no effect on glial activation (data not shown). Moreover, analysis for microglia morphology showed that CAP significantly restored the MPTP-induced decrease in length of the microglia branch (increase in microglial activation) in the SN. This CAP effect on microglia morphology was reversed by treatment with AM251 and AM630 in vivo (Supplementary Figures 1(a) and 1(b)).

### 3.3. Anandamide Protects Nigrostriatal Dopamine Neurons from MPTP Neurotoxicity by Preventing Brain Inflammation and Gliosis *In Vivo*

Several lines of evidence have shown that TRPV1 activation by CAP produces anandamide (AEA), which activates TRPV1 and/or the CB receptor [15, 24, 25] and prevents neurodegeneration *in vivo* and *in vitro* [16, 26–28]. Accordingly, we determined whether AEA, when exogenously administered, could rescue nigrostriatal dopamine neurons. Mice intraperitoneally received AEA (0.5 mg/kg) for 1 day, starting at 12 hours after the last MPTP injection or PBS as a control (Figure 2(a)). The results of TH immunohistochemistry showed that MPTP caused the degeneration of nigrostriatal dopamine neurons, compared to PBS (Figures 2(b) and 2(c)). In MPTP-lesioned mice, posttreatment with AEA rescued TH^+^ dopamine neurons in the SN *in vivo* (Figures 2(b) and 2(d)) and TH^+^ fibers in the STR (Figures 2(c) and 2(d)) compared to MPTP-lesioned vehicle-treated mice. When quantified and expressed as a percentage of TH^+^ neurons in the SN or TH^+^ fibers in the STR of MPTP-lesioned mice, AEA was found to increase the number of TH^+^ neurons by 41% (Figure 2(d); *P* < 0.001) and the optical density of TH^+^ fibers by 36% (Figure 2(d); *P* < 0.001). AEA alone had no effects on the number of TH^+^ neurons in the SN or TH^+^ fibers in the STR (Figure 2(d)).

**Figure 5:**
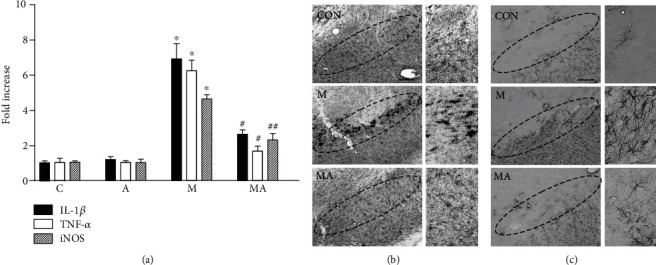
Anandamide inhibits glial activation and production of proinflammatory molecules in the SN of MPTP-treated mice in vivo. Mice were intraperitoneally given an injection of PBS as a control (CON) or MPTP only (M) or MPTP + anandamide (0.5 mg/kg; MA) 12 hours after the last injection of MPTP. (a) Real-time PCR showing the messenger RNA expression of proinflammatory molecules in the SN. The total RNA was isolated from the SN at 1 day after the last injection of MPTP or vehicle in the absence or presence of anandamide (refer to Figure 2(a)). These results are means ± SEM of four samples. C: control; A: anandamide; M: MPTP; MA: MPTP and anandamide. ^∗^*P* < 0.01, significantly different from C. ^#^*P* < 0.01 and ^##^*P* < 0.05, significantly different from M (one-way ANOVA with the Neuman-Keuls post hoc test). (b, c) Photomicrographs of CD11b^+^ microglia and GFAP^+^ astrocytes in the SN of MPTP-treated mice in vivo. Mice that received PBS as a control (CON), MPTP (M), and MPTP and anandamide (MA) were sacrificed at 3 days after the last MPTP injection (refer to Figure 2(a)). Brains were removed, and coronal sections (30 *μ*m) were cut using a sliding microtome. Every sixth serial section was selected and immunostained with CD11b antibody for microglia (b) or GFAP antibody for astrocytes (c). These data are representative of five to six animals per group. Dotted lines indicate SNpc. Scale bars: 300 *μ*m (b); 250-420 *μ*m (c).

We next determined whether AEA neuroprotection on dopamine neurons could be associated with MPTP-induced expression of inflammatory cytokines in vivo. The expression of the mRNA levels of proinflammatory molecules was evaluated in animals receiving PBS, MPTP, and MPTP + AEA 1 day after the last MPTP injection. The results of RT-PCR showed that the mRNA levels of IL-1*β*, TNF-*α*, and iNOS were significantly increased in the SN of MPTP-lesioned mice compared with PBS-treated SN (*P* < 0.01; Figure 5(a)). Treatment with AEA reduced MPTP-induced increases in expression of IL-1*β* by 83% (*P* < 0.01; Figure 5(a)), TNF-*α* by 90% (*P* < 0.01; Figure 5(a)), and iNOS by 56% (*P* < 0.05; Figure 5(a)) in the SN. AEA alone had no effects on the mRNA levels of IL-1*β*, TNF-*α*, or iNOS.

Next, we analyzed the effects of AEA on glial activation by immunostaining with CD11b and GFAP in the SN 3 days after the last MPTP injection (Figures 2(a) and 5(b)). The majority of CD11b^+^ microglia displayed an activated morphology, including larger cell bodies with short processes in the SN of MPTP-lesioned mice (Figure 5(b), left panel) compared to resting morphology in PBS-treated control (Figure 5(b)). Treatment with AEA dramatically attenuated the number of CD11b^+^-activated microglia in the MPTP-lesioned SN (Figure 5(b)). This inhibitory effect of AEA on microglial activation was also confirmed by analysis of microglia morphology showing that AEA significantly restored the MPTP-induced decrease in the length of the microglia branch in the SN (Supplementary Figures 2(a)and 2(b)). Astrocytes also exhibited reactive morphology with thick processes (Figure 4(c)) in the MPTP-lesioned SN as determined by GFAP immunohistochemical staining. Treatment with AEA profoundly mitigated GFAP^+^-reactive astrocytes in the MPTP-lesioned SN (Figure 5(b), right panel). A few of the GFAP^+^ astrocytes were observed in the SN of PBS-treated mice (Figure 5(b)).

## 4. Discussion

The main findings of the present study are the existence and effects of the *in vivo* functional crosstalk between TRPV1 and the CB receptors, rescuing nigrostriatal dopamine neurons in the MPTP mouse model of PD. TRPV1-activated neuroprotection by capsaicin in the MPTP mouse is inhibited by antagonizing the CB1 receptor or the CB2 receptor. Capsaicin-induced inhibition of both glial activation and production of inflammatory mediators was also abolished by the treatment of CB antagonists. In addition, activation of two receptors (TRPV1 and CB receptor) by AEA showed the neuroprotective effects on the nigrostriatal dopamine neurons by inhibiting glial activation and production of inflammatory mediators in the MPTP mouse model of PD.

Neuroinflammation is considered the major neuropathological feature in neurodegenerative disorders including PD, Alzheimer's disease, frontal temporal dementia, and amyotrophic lateral sclerosis. Under neurodegenerative conditions, both microglia and astrocytes transformed to reactive phenotypes and participated in the production of inflammatory mediators in the central nervous system [29]. In the animal model of PD and in PD patients, reactive microglia/astrocytes (intense CD11b/GFAP immunoreactivity and hypertrophy) and increased level of proinflammatory mediators exist in the SNpc and STR, indicating the possible involvement of gliosis-derived inflammatory processes in PD [3, 5, 7, 30]. Many experimental studies have shown that inhibition of glial activation-derived inflammatory response contributes to a protection of dopamine neurons *in vivo* and *in vitro* [3, 5, 31, 32]. Recent reports including ours show that the activation of TRPV1 by CAP [10, 11, 13] or the CB receptor by CB1/2 agonists [18, 21, 33] can prevent glial activation, oxidative stress, and expression of proinflammatory molecules *in vivo* in animal models of PD produced by the administration of MPTP, 6-OHDA, and LPS. These results are in line with the present data that TRPV1 activation by CAP or the activation of both TRPV1 and the CB receptor by AEA inhibits glial activation and production of proinflammatory mediators in MPTP-lesioned SN *in vivo*. Taken together, the present data suggest that the neuroprotective effect of CAP and AEA is associated with the property of TRPV1 and the CB receptor to block glial activation and production of inflammatory molecules in the MPTP mouse model of PD.

Anandamide (AEA) is an endogenous ligand for both TRPV1 and the CB receptor and modulates the endocannabinoid system in the central nervous system [8, 9, 17]. Pisani et al. reported that the level of AEA is increased in the cerebrospinal fluid of PD patients [34]. Molecular imaging studies reveal that the density of the CB1 receptor is increased in the putamen of PD patients and MPTP-treated marmosets, indicating an association of the endocannabinoid system with PD progression [35, 36]. Indeed, an increase in the levels of AEA by inhibiting fatty acid amide hydrolase (a degradation enzyme for AEA) enhanced TH immunoreactivity in the SN and STR and suppressed microglial activation in MPTP-treated mice [37]. This is in line with our data showing that exogenous delivery of AEA attenuates neurotoxicity and glial activation-derived inflammatory responses in MPTP-lesioned mice. Collectively, these results suggest that the upregulation of endocannabinoids might be implicated with the compensatory response which is aimed at reducing the loss of dopamine neurons and/or neuroinflammation in the animal model of PD and possibly in PD patients.

Several lines of evidence have shown that TRPV1 and CB receptors are widely colocalized in different types of neurons of the several brain areas including dopamine neurons in the SN [15, 38, 39]. Numerous studies including ours reported functional associations between TRPV1 and CB receptor, which is either neurotoxic [15, 40, 41] or neuroprotective [8, 16, 17] *in vitro* and *in vivo*. Regarding this, the present study demonstrates that the CB1 and CB2 receptor antagonists inhibit CAP-induced reduction in MPTP toxicity against dopamine neurons, glial activation, and mRNA expression of proinflammatory molecules, indicative of functional interactions between TRPV1 and CB receptors. This beneficial effect might result from molecules, which are synthesized by CAP-activated TRPV1 and are able to activate these two receptors (TRPV1 and CB receptors). Among them, AEA, an endogenous ligand for both TRPV1 and CB receptors, is synthesized by CAP-activated TRPV1 and activates both TRPV1 and CB receptors resulting in the neuroprotection in ouabain-induced excitotoxicity [16] and inhibition of locomotor activity *in vivo* [25]. The result of the present study shows that exogenous delivery of AEA attenuates MPTP neurotoxicity and glial activation-derived inflammatory responses. Taken together, our data carefully suggest that AEA synthesized by CAP-activated TRPV1 appears to inhibit glial activation-derived neuroinflammation and the resultant survival of dopamine neurons in the MPTP mouse model of PD, although we did not provide any direct evidence of AEA synthesis by CAP-activated TRPV1.

MPTP is metabolized by astrocytic monoamine oxidase B (MAO-B) to 1-methyl-4-phenylpyridine (MPP^+^), which is uptaken into dopamine neurons and then eventually leads to dopamine neuronal death [18, 19, 42]. Regarding this, we have shown that conversion of MPTP into MPP^+^ is almost completed at 12 hours after the last MPTP injection [18, 19] and TRPV1 activation by CAP and CB receptor agonists does not interrupt the conversion of MPTP into MPP^+^*in vivo* [11, 18]. Thus, it can rule out the possibilities that the observed neuroprotection by delayed treatment with AEA (12 hours after the last MPTP injection) might be attributable to reducing the metabolism of MPTP to MPP^+^ or preventing MPP^+^ uptake into dopamine neurons.

Finally, the present study suggests a novel neuroprotective mechanism for dopamine neurons, resulting from the crosstalk between TRPV1 and CB receptors in the MPTP mouse model of PD. The activation or interaction of TRPV1 and CB receptors may be beneficial for regulating the glial activation and production of proinflammatory mediators, resulting in an increased survival of dopamine neurons in MPTP-lesioned mice.

## 5. Conclusion

Finally, the present study suggests a novel neuroprotective mechanism for dopamine neurons, resulting from the crosstalk between TRPV1 and CB receptors in the MPTP mouse model of PD. The activation or interaction of TRPV1 and CB receptors may be beneficial for regulating the glial activation and production of proinflammatory mediators, resulting in an increased survival of dopamine neurons in MPTP-lesioned mice. Thus, the activation of both TRPV1 and CB receptors by compounds related to the endovanilloid/endocannabinoid system might constitute a new therapeutic strategy to treat PD.

## Data Availability

The data used to support the findings of this study are all provided within the article.

## Supplementary Materials

Supplementary 1 Supplementary Figure 1: crosstalk between CB and TRPV1 inhibits microglial activation in the SN of MPTP-treated mice in vivo. (a) The skeletonized images are processed using ImageJ with Skeleton plugin in Figure 4(b). (b) Quantification results of microglia process length in each animal groups. Bars represent the means ± SEM of five to six animals per group. ^∗^*P* < 0.001, significantly different from control. ^&&^*P* < 0.01, significantly different from MPTP. ^#^*P* < 0.001 and ^##^*P* < 0.01, significantly different from MPTP and CAP (one-way ANOVA with the Neuman-Keuls post hoc test).

Supplementary 2 Supplementary Figure 2: anandamide suppresses microglial activation in the SN of MPTP-treated mice in vivo. (a) The skeletonized images are processed using ImageJ with Skeleton plugin in Figure 5(b). (b) Quantification results of microglia process length in each animal groups. Bars represent the means ± SEM of five to six animals per group. ^∗^*P* < 0.001, significantly different from control. ^&^*P* < 0.001, significantly different from MPTP (one-way ANOVA with the Neuman-Keuls post hoc test).
