# Supplementary figures and images for: Functional Crosstalk between CB and TRPV1 Receptors Protects Nigrostriatal Dopaminergic Neurons in the MPTP Model of Parkinson's Disease
Source: J Immunol Res. 2020 Sep 28;2020:5093493. doi: 10.1155/2020/5093493 (PMC7539109; doi:10.1155/2020/5093493)

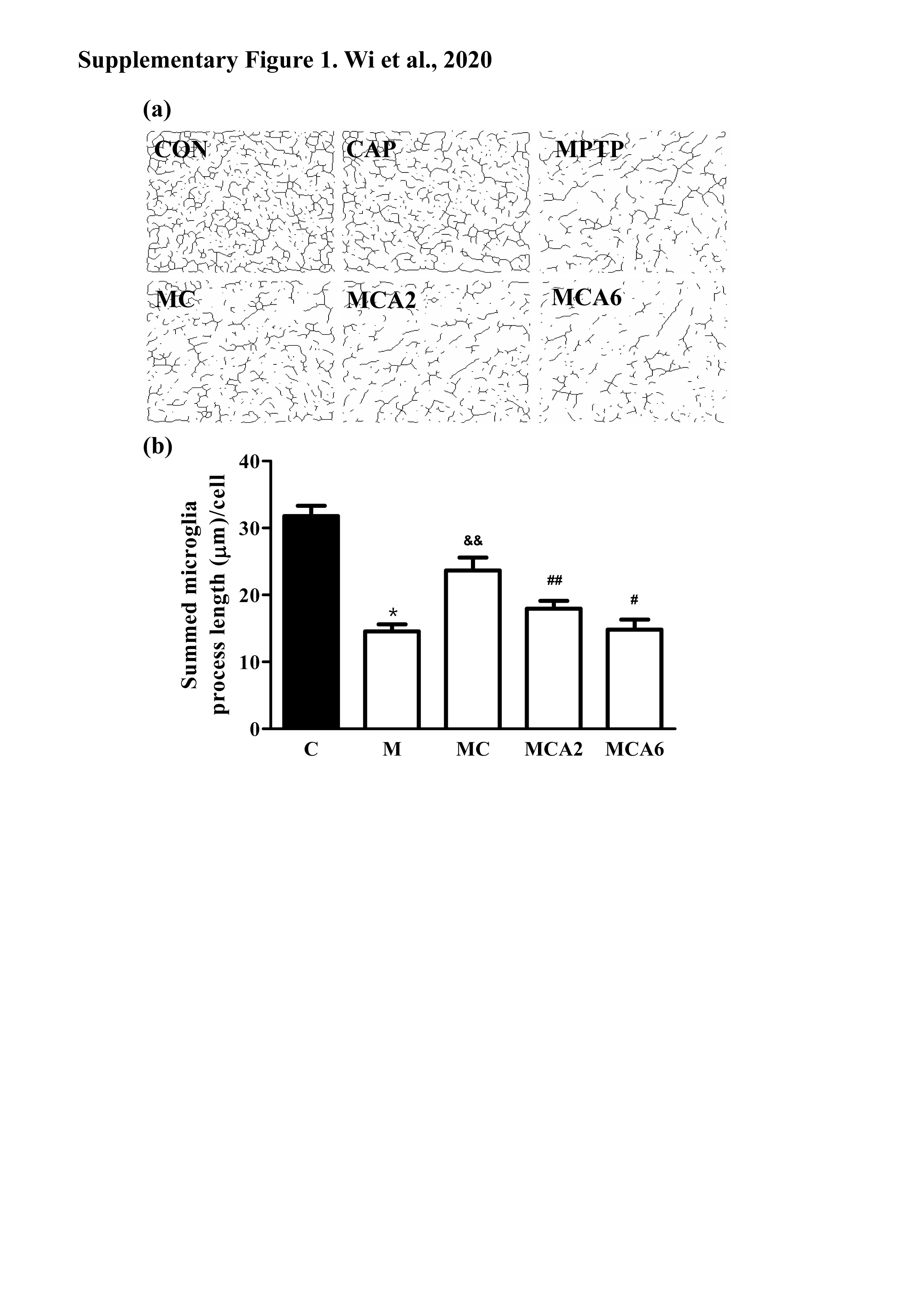

Supplement: Supplementary 1 — Supplementary Figure 1: crosstalk between CB and TRPV1 inhibits microglial activation in the SN of MPTP-treated mice in vivo. (a) The skeletonized images are processed using ImageJ with Skeleton plugin in Figure 4(b). (b) Quantification results of microglia process length in each animal groups. Bars represent the means ± SEM of five to six animals per group. ∗P < 0.001, significantly different from control. &&P < 0.01, significantly different from MPTP. #P < 0.001 and ##P < 0.01, significantly different from MPTP and CAP (one-way ANOVA with the Neuman-Keuls post hoc test). [file 5093493.f1.jpg]

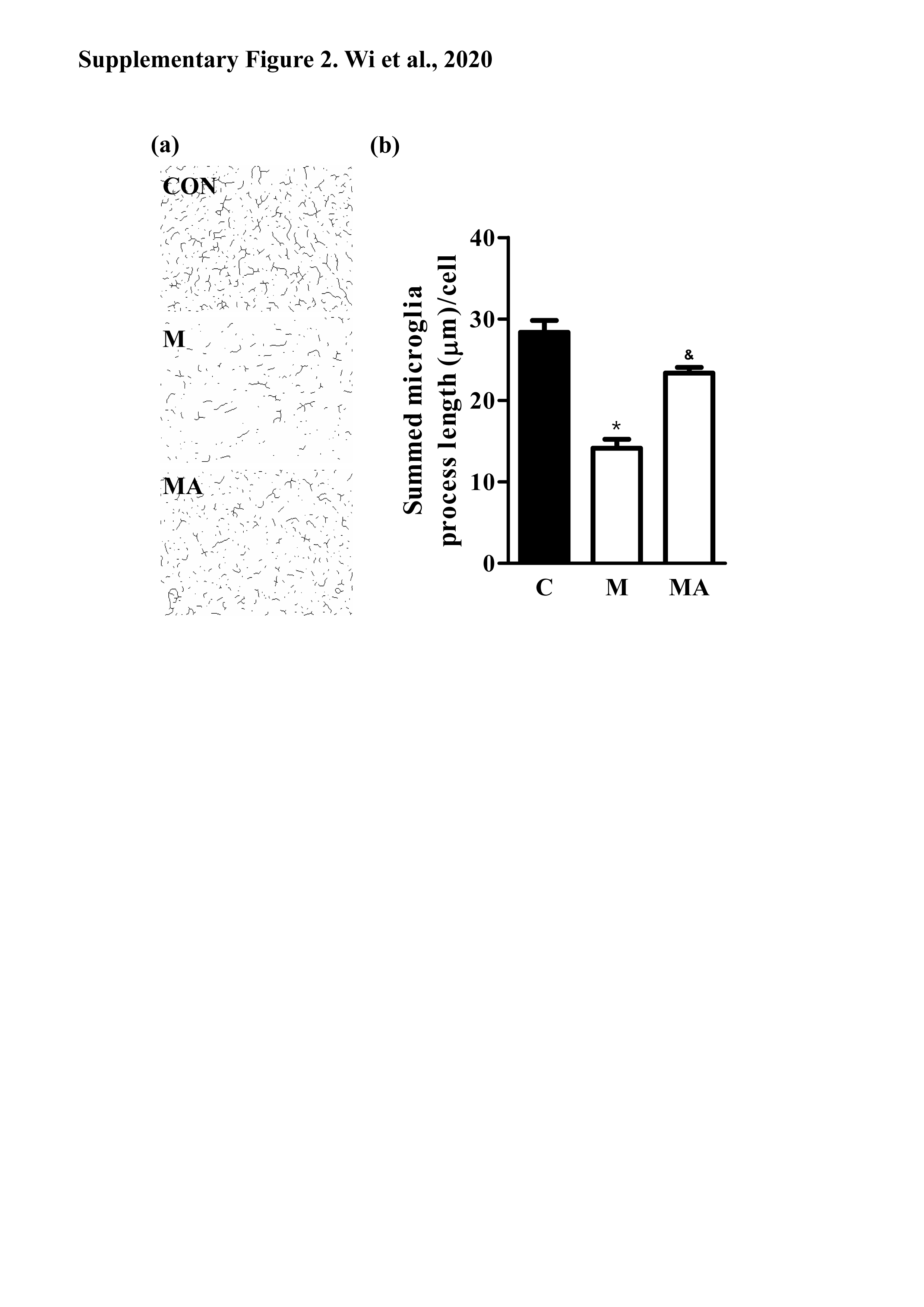

Supplement: Supplementary 2 — Supplementary Figure 2: anandamide suppresses microglial activation in the SN of MPTP-treated mice in vivo. (a) The skeletonized images are processed using ImageJ with Skeleton plugin in Figure 5(b). (b) Quantification results of microglia process length in each animal groups. Bars represent the means ± SEM of five to six animals per group. ∗P < 0.001, significantly different from control. &P < 0.001, significantly different from MPTP (one-way ANOVA with the Neuman-Keuls post hoc test). [file 5093493.f2.jpg]
